# *Ehretia* Species Phytoconstituents as Potential Lead Compounds against *Klebsiella pneumoniae* Carbapenemase: A Computational Approach

**DOI:** 10.1155/2023/8022356

**Published:** 2023-10-12

**Authors:** Samson O. Oselusi, Nicole R. S. Sibuyi, Mervin Meyer, Abram M. Madiehe

**Affiliations:** ^1^Nanobiotechnology Research Group, Department of Biotechnology, University of the Western Cape, Private Bag X17, Bellville, Cape Town 7535, South Africa; ^2^DSI/Mintek Nanotechnology Innovation Centre (NIC), Biolabels Research Node, Department of Biotechnology, University of the Western Cape, Private Bag X17, Bellville, Cape Town 7535, South Africa

## Abstract

The evolution of antibiotic-resistant carbapenemase has negatively impacted the management of critical healthcare-associated infections. *K. pneumoniae* carbapenemase-2- (KPC-2-) expressing bacteria have developed resistance to conventional therapeutic options, including those used as a last resort for life-threatening diseases. In this study, *Ehretia* species phytoconstituents were screened for their potential to inhibit KPC-2 protein using *in silico* approaches. Molecular docking was used to identify strong KPC-2 protein binding phytoconstituents retrieved from the literature. The best-docked conformation of the ligands was selected based on their glide energy and binding interactions. To determine their binding free energies, these hit compounds were subjected to molecular mechanics with generalized born and surface area (MM-GBSA) in the PRIME module. Pharmacological assessments of the ligands were performed to evaluate their drug-likeness. Molecular dynamic (MD) simulations were used to analyze the conformational stability of the selected druglike compounds within the active site of the KPC-2 protein. Overall, a total of 69 phytoconstituents were compiled from the literature. Fourteen of these compounds exhibited a stronger binding affinity for the protein target than the reference drugs. Four of these top hit compounds, DB09, DB12, DB28, and DB66, revealed the highest efficacy in terms of drug-likeness properties. The MD simulation established that among the druglike compounds, DB66 attained stable conformations after 150 ns simulation in the active site of the protein. We concluded that DB66 from *Ehretia* species could play a significant role in therapeutic efforts against KPC-2-expressing bacteria.

## 1. Introduction

Antimicrobial resistance (AMR) is a natural process by which microorganisms develop mechanisms that protect them from the effects of drugs to which they were previously sensitive [1]. The emergence and global spread of antimicrobial-resistant pathogens pose a great challenge to medicine and public health [2]. AMR is currently estimated to cause 700 000 deaths globally each year. This is projected to force more than 24 million people into extreme poverty by 2030 and kill more than 10 million per annum by 2050 [3]. As part of its strategies to fight this threat, the World Health Organization (WHO) in 2017 released a global priority list of antibiotic-resistant pathogens to guide drug discovery and research [4]. This list contains 12 bacterial strains that are categorized into critical, high, and medium priority. The need for new and effective therapeutic interventions against carbapenemase-expressing pathogens, such as *Acinetobacter baumannii*, *Pseudomonas aeruginosa*, and *K. pneumoniae*, was also stressed [4, 5]. *K. pneumoniae*, a member of the Enterobacteriaceae family, is an opportunistic nosocomial or hospital-acquired pathogen known for its broad spectrum of diseases and antibiotic resistance [6]. *K. pneumoniae* accounts for about one-third of all Gram-negative infections related to the respiratory and urinary tracts, surgical wounds, and life-threatening infections such as septicaemia and endocarditis [7, 8].

The incidence of *K. pneumoniae* is escalated by the ability of the bacterium to acquire resistance to all major antibiotics that were used to kill or control them [9]. Of concern is the rate at which they acquire virulent genes to become increasingly endemic in different parts of the world. For instance, a recent study reported the outbreak of two phenotypes of *K. pneumoniae* with different virulence in Southwest China [10]. Another study also established an outbreak of a novel *K. pneumoniae* strain, New Delhi Metallo-*β*-lactamase in a Peruvian healthcare facility among patients that were admitted for the diagnosis and treatment of COVID-19 [11]. Furthermore, KPC-2, a member of Ambler class A carbapenemase, has emerged as a leading cause of healthcare-associated infections, known for its hydrolytic activity against a broad spectrum of substrates including carbapenem and most other *β*-lactam antibiotics [12, 13]. KPC-2 was discovered and named after carbapenem-resistant *K. pneumoniae* in 1996, and its sequence variants were later identified in various pathogenic Gram-negative bacteria such as *E*. *coli*, *Enterobacter cloacae*, *A. baumannii*, and *P. aeruginosa* [12]. Drugs for diseases associated with these pathogens are often degraded by carbapenemases, resulting in treatment failure. Furthermore, the outbreaks and spread of KPC-2 strains within healthcare facilities have been reported in various parts of the world [14–22]. KPC-2-expressing bacteria have significantly limited their antibiotic options to drugs such as polymyxins and fosfomycin which were previously not considered ideal because of efficacy and toxicity problems [23]. Therefore, the search for effective and more reliable inhibitors against KPC-2 protein could open the way for the development of new antimicrobial agents.

Plants have been successfully used for centuries in the fight against chronic and infectious diseases, and they continue to present ideal sources of novel antimicrobial agents for multidrug-resistant bacteria. Examples of these medicinal plants have been described in various studies [21, 24]. The genus *Ehretia* belongs to the Boraginaceae family with more than 150 species distributed as trees and shrubs across the tropical and subtropical regions of Asia, Africa, Europe, Australia, and North America [25]. *Ehretia* species, such as *E. laevis*, *E. rigida*, *E. acuminata*, *E. longiflora*, *E. buxifolia*, *E. microphylla*, and *E. obtusifolia*, among others, have been broadly used in herbal medicine for the treatment of diseases, including those associated with the respiratory (pneumonia, asthma, and cough), endocrine (diabetes mellitus), and digestive (diarrhea, jaundice, dysentery, ulcers) systems [26]. Phytochemical screening of various solvent extracts from *Ehretia* species has established that the plant's barks, fruits, roots, and leaves contain health-beneficial phytoconstituents. The phenolic acids, flavonoids, fatty acids, alkaloids, tannins, and benzoquinones have been isolated from this plant and reported to have various degrees of biological activities including, antitubercular, antisnake venom, and antibacterial [25, 26]. For instance, ehretianone, obtained from the root bark of *E. buxifolia* has antisnake venom properties, while ehretiolide and prenylhydroquinone from the root of *E. longiflora* possess antitubercular activity [25, 27]. Likewise, rutin has shown strong inhibitory activity against pathogens such as *Klebsiella* sp., *Staphylococcus aureus*, *A. baumannii*, and *P. aeruginosa* [28]. The antimicrobial properties of riboflavin against pathogenic microbes such as *S. aureus*, *Enterococcus faecalis*, *K. pneumonia*, and *P. aeruginosa*, among others, have also been reported [29]. To the best of our knowledge, no *study has been* conducted to investigate the prospects of *Ehretia* species phytoconstituents in drug discovery against KPC-2 enzymes.

The preclinical stage of drug discovery is a tedious, time-consuming, and expensive process. At this stage, the selection of lead compounds is performed by screening a library of potential drugs for their efficacy through target identification, pharmacokinetic analyses, and toxicity profiling [30–32]. Recent advances in drug discovery have allowed the use of computational approaches such as molecular docking, MD simulations, and drug-likeness prediction, among others to provide cost-effective and efficient alternatives for finding potential drug candidates during the early stages of drug discovery [32–34].

Motivated by the antimicrobial properties of *Ehretia* species, as well as the application of computational techniques in drug discovery, this study explored the *in silico* binding potential of *Ehretia* phytoconstituents with KPC-2 protein. Hence, the results could be used to obtain relevant information before conducting appropriate *in vitro* and *in vivo* experiments. The study could also provide insight into the full potential of available natural products in the search for effective and efficient novel therapeutic interventions against KPC-2-expressing bacteria.

## 2. Materials and Methods

### 2.1. Data Collection

A literature search was conducted to retrieve phytoconstituents of *Ehretia* species. The search was performed on freely available public databases which include Google Scholar, Scopus, and PubMed, using the keywords, “Phytoconstituents from *Ehretia* species,” and “Natural compounds from *Ehretia* species.” The simplified molecular-input line-entry system (SMILES) structures of the phytoconstituents were obtained from the PubChem database (http://pubchem.ncbi.nlm.nilh.gov) and saved as a txt file. The crystal structure of KPC-2 beta-lactamase complexed with hydrolyzed faropenem at a resolution of 1.40 Å was retrieved from the RCSB Protein Data Bank (PDB ID: 5UJ4) database (https://www.rcsb.org) and used as the protein target.

### 2.2. Testing the Reliability of the Docking Protocol

The exactness and reliability of the docking protocol were substantiated according to previously described methods [35, 36]. The cocrystalized ligand (coligand) of the resolved protein structure, as found in the PDB (http://www.pdb.org/pdb), was separated and saved as an SDF file. Thereafter, the ligand was docked into the protein's active site using the Maestro Schrödinger suite (Schrödinger, LLC, New York, NY, 2021-4). PyMOL molecular visualizer was used to superimpose the docked complex against the experimentally resolved protein that is harboring its native ligand to generate the root-mean-square deviation (RMSD) value.

### 2.3. Molecular Docking

#### 2.3.1. Ligand Preparation

Sixty-nine phytoconstituents of *Ehretia* species were collated from published literature, and they were used to create a library of compounds. The text file of these phytoconstituents was first converted to Spatial Data File (SDF) using Data Warrior software (v 5.5.0). The LigPrep module in the Maestro Schrödinger suite was used to set up and start the ligand preparation step. LigPrep uses sophisticated rules to convert structure from 2D to 3D by adding hydrogens and considering bond lengths or angles based on the correct chiralities, tautomers, and ring conformations in the ligands which helps to reduce downstream computational errors [37]. The possible protonation states were set at a physiological pH range of 7 ± 2 with the help of the Epik ionization program [38], and the OPLS4 force field was used for energy minimization [39, 40]. All other parameters on the LigPrep interface were kept as default. Three standard inhibitors of KPC-2 proteins, viz relebactam, avibactam, and vaborbactam [41, 42], and the coligand (faropenem) was used as a reference drug.

#### 2.3.2. Protein Preparation

The crystal structure of the KPC-2 protein (PDB ID: 5UJ4) from *K. pneumoniae* was prepared to ensure structural correctness before docking simulation. This step was performed using the Protein Preparation Wizard of Schrödinger Maestro [43]. Polar hydrogen atoms were added, water molecules beyond 5 Å from het groups were removed, charges were defined at default pH (7 ± 2) using Epik [38], and bond orders were assigned. Finally, restrained minimization was carried out using an OPLS4 force field to alleviate steric clashes while heavy atoms converged at a predefined RMSD value of 0.30 Å [37, 44].

#### 2.3.3. Grid Generation and Molecular Docking

The receptor grid generation panel in Maestro was used to define the area around the active site of the prepared protein. The grid box resolution was centered around the coligand faropenem at coordinates 21.36, 17.59, and 8.29 corresponding to the *x*, *y*, and *z* axes, respectively. Next, the molecular docking simulation job was set up on the ligand docking panel of the Glide module by selecting extra precision (XP) mode [45] and keeping all other parameters at default. The output was submitted to a remote cluster machine (https://users.chpc.ac.za/), where the docking calculations were performed. The binding energy values (kcal/mol) were obtained, and the docked conformation with the lowest docking score was identified for each ligand. Finally, all phytoconstituents with more promising affinity than the reference drugs were selected for analysis.

#### 2.3.4. Binding Free Energy Calculations

To estimate the absolute free energies of the protein-ligand interactions, MM-GBSA calculations in the Prime module [43] of the Schrödinger Maestro suite were used for rescoring the protein-ligand complex. The surface generalized born (SGB) model, the variable dielectric (VD), and the OPLS4 force field were selected, keeping all other parameters constant [46, 47].

### 2.4. Drug-Likeness and Pharmacokinetic Properties

The SDF format of the selected compounds was uploaded onto the SwissADME web server [48], ADMElab [49], and Osiris DataWarrior [50] to calculate the physicochemical and pharmacokinetic properties of the phytoconstituents. The rule-based molecular descriptors such as Lipinski's rule of five (Ro5) and Veber were used to estimate the drug-likeness of the compounds, according to Oselusi et al. [31]. The pharmacokinetic prospects of the natural compounds were estimated in terms of absorption, distribution, metabolism, excretion, and toxicity (ADMET) properties.

### 2.5. Molecular Dynamics (MD) Simulation

To determine the stability of the docking interactions, 150 ns-long MD simulations were carried out on the protein-ligand complexes and compared with the best reference drug. This was performed using the Desmond module of the Schrödinger Maestro suite with the OPLS4 force field according to the method described by Choudhary et al. [51]. Each of the selected protein-ligand interactions and the unbound protein was solvated with a predefined three-site transferable intermolecular potential (TIP-3P) water model, and the boundary condition was made an orthorhombic box shape. The overall charge of the system was neutralized by adding Na^+^ and Cl^−^ ions with a salt concentration of 0.15 M to mimic physiological conditions. To ensure that there were no steric clashes or incorrect geometry in the system, the box volume was further minimized by implementation of the hybrid method of steepest descent and Limited-memory Broyden-Fletcher-Goldfarb-Shanno (LBFGS) [52]. The simulations were made under the isothermal-isobaric (NPT) ensemble, where temperature and the atmospheric pressure of the system were kept constant at 310 Kelvin and 1.013 bar, respectively, with an energy of 1.2. The job was written out and submitted to run on a remote cluster machine (https://users.chpc.ac.za/). Finally, the simulation interaction diagram (SID) tool of Maestro Schrödinger was used to further analyze the trajectories generated after a successful simulation. Finally, the stability of the protein-ligand interactions was explored through the calculation of RMSD and root-mean-square fluctuation (RMSF), the radius of gyration (rGyr), hydrogen bond interactions, and solvent accessible surface area (SASA).

## 3. Results

### 3.1. Molecular Docking and MM-GBSA Calculations

**Figure 1:**
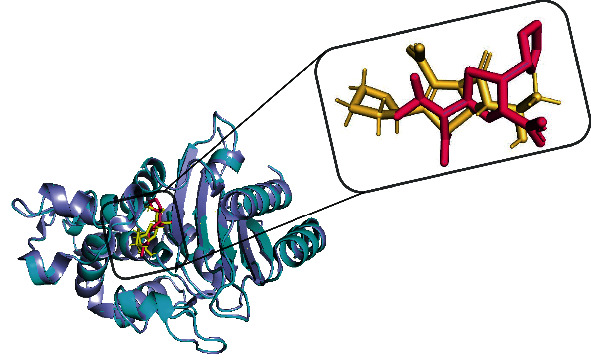
Validation of the docking protocol by superimposing the coligand (pink) and after redocking the same ligand (yellow) exhibited an RMSD of 0.157 Å.

A total of 69 phytoconstituents were identified from the literature. They belong to the classes of phenolic acids, flavonoids, alkaloids, fatty acids, vitamins, benzoquinones, essential oils, pentacyclic triterpenoids, and glycosides (Table S1). The 3 selected inhibitors of *K. pneumoniae* and the coligand that were used as controls are also described in Table S1. For validation of the docking protocol, the coligand was removed and docked in the protein binding site. The coligand and after redocking the same ligand are superimposed as shown in Figure 1, exhibiting an RMSD value of 0.157 Å.

**Figure 2:**
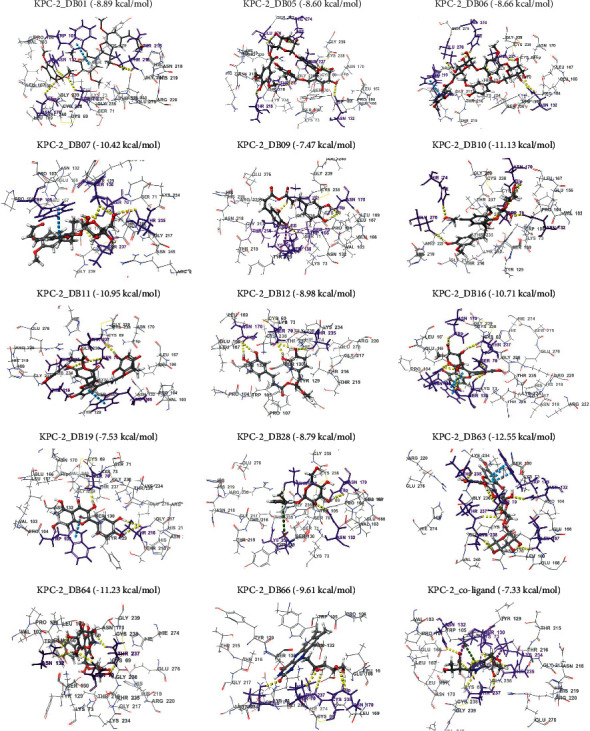
Binding interactions of the top *Ehretia* species phytoconstituents, and the coligand to KPC-2. The amino acid residues within the binding sites are presented, and the ligands are coloured by elements. Yellow dotted lines represent hydrogen bond interactions, and purple, green, and blue dotted lines represent salt bridge, *π*-cation, and *π*-*π* stacking bonds, respectively. All amino acids that participated in the interactions are coloured orange.

Molecular docking interactions of all the compounds in the active site of KPC-2 protein were analyzed to determine the binding affinities using several parameters, including a Glide-based docking score, hydrogen bond interaction, *π*-cation, *π*-*π* stacking interaction, and salt bridge formation. The docking scores of the compounds ranged from -12.55 to 0.89 kcal/mol (Table S1). As shown in Table S1, among the reference drugs (relebactam, avibactam, vaborbactam, and faropenem), the coligand (faropenem) had the highest energy score (-7.33 kcal/mol). The coligand showed 6 hydrogen bond interactions with Ser70, Ser130, Asn132, Thr235, and Thr237 (Figure 2). The atoms of this molecule also presented 2 salt bridge interactions with Lys73, and Lys234 and a *π*-cation interaction with Lys73. The 14 phytoconstituents that showed comparable binding energies to that of the coligand were selected for further analysis.

Among the phytoconstituents, DB63 exhibited the highest binding energy (-12.55 kcal/mol) presenting 7 hydrogen bonding interactions with Ser70, Asn132, Thr235, Thr237, Cys238, and Leu167 and 3 *π*-*π* stacking bonds with Trp105. DB64 and DB10 exhibited binding energies of -11.23 and -11.13 kcal/mol, respectively (Figure 2). Additionally, DB64 produced 5 hydrogen bonding interactions viz, Ser70, Asn132, and Thr237 while DB10 interacts via 7 hydrogen bonds involving Ser70, Asn132, Asn170, Hie274, and Glu276. Similarly, DB11, DB16, and DB07 displayed comparable binding energies of -10.95, -10.71, and -10.42 kcal/mol, respectively. The binding interactions of DB11 revealed the formation of 4 hydrogen bonds involving Ser70, Thr216, Thr235, and Thr237, and an additional *π*-*π* stacking bond with Trp105. The atoms of DB16 participated in the formation of 7 hydrogen bond interactions with Ser70, Ser130, Asn132, Asn170, and Thr237. DB16 also produced 3 *π*-*π* stacking bonds with Trp105. The compound, DB07, exhibited 6 hydrogen bond interactions with Ser70, Ser130, Thr235, and Thr237 and an additional *π*-*π* stacking bond with Trp105. The atoms of DB66 revealed a binding energy of -9.61 kcal/mol, presenting 5 hydrogen bonding interactions with Ser70, Asn170, Thr235, Thr237, and Cys238. Other phytoconstituents, such as DB12, DB01, DB28, DB06, and DB05, showed comparable binding energies of -8.98, -8.89, -8.79, -8.66, and -8.60 kcal/mol, respectively. Analyses of the binding interactions of DB12 showed that it interacts via 5 hydrogen bonds with Ser70, Asn170, and Thr235. Similarly, five residues comprising Ser70, Asn132, Asn170, Thr215, and Thr216 established 5 hydrogen bond interactions with DB01. DB01 also formed *π*-*π* stacking bond with Trp105. DB28 formed 3 hydrogen bond interactions with Asn132, Asn170, and Thr237 while DB06 formed 7 hydrogen bonds with Asn132, Asn218, Thr237, Hie274, and Glu276, and a *π*-*π* stacking with His219. Similarly, DB05 interacts via 7 hydrogen bonds with Asn132, Thr215, Thr235, Thr237, Hie274, and Glu276. DB19 and DB09 displayed comparable binding energy with the coligand. DB19 formed 3 hydrogen bond interactions with Ser70, and Thr216, and *π*-*π* stacking bond with Trp105 (Figure 2). Finally, apart from the 4 residues (Ser130, Asn170, Thr235, and Thr237) that produced 5 hydrogen bond interactions, Lys234 also exhibited a salt bridge interaction with DB09.

The binding details of these top hit *Ehretia* species phytoconstituents and the coligand are summarized in Table 1.

The predicted binding free energy (MM-GBSA dG Bind) for the phytoconstituents ranged from -79.36 to -44.79 kcal/mol (Table 1). DB11 had the maximum affinity for the KPC-2 protein, followed by DB63 and DB66 with dG Bind of -79.36, -66.33, and -64.63 kcal/mol, respectively. In contrast, the coligand had a free energy value of -14.30 kcal/mol.

**Figure 3:**
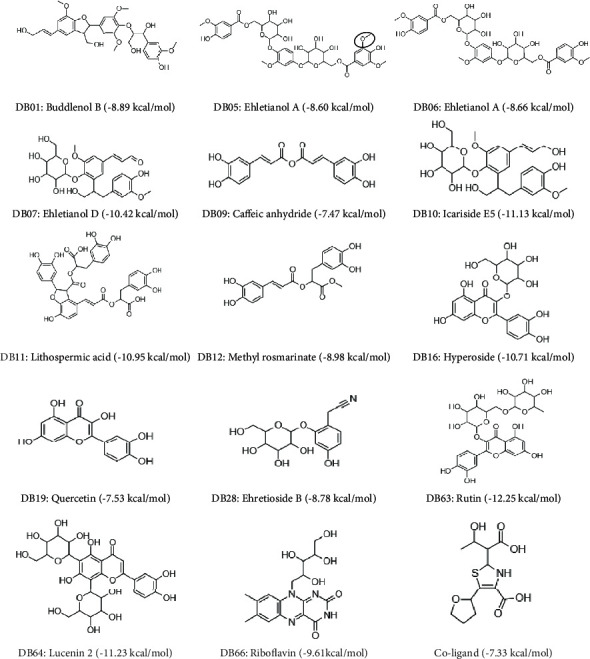
2D structure of the 14 top hit *Ehretia* species phytoconstituents that displayed a higher binding affinity for KPC-2 protein. The structural difference between DB05 and DB06 is highlighted.

The chemical structures of the *Ehretia* species phytoconstituents and coligand described above are shown in Figure 3. Only DB05 and DB06 are structurally similar except for the presence of an additional methoxy functional group (O-CH_3_) in one of the benzene rings of DB05, which resulted in significant effects on the binding behaviour of the phytoconstituents.

### 3.2. Physicochemical Properties, Drug-Likeness, Pharmacokinetic, and Toxicity Profiling

The physicochemical, drug-likeness, and toxicity properties of the selected *Ehretia* species phytoconstituents are profiled as displayed in Table 2. The molecular weight of the hit compounds ranges from 302.24 to 794.71 g/mol. The hydrogen bond acceptor (HBA) and hydrogen bond donor (HBD) values range from 7 to 20 and 4 to 12, respectively. All the tested compounds showed good aqueous solubility (Log*S*) with values ranging from -3.93 to -1.254. Similarly, the lipophilicity (Log*P*) values range between -0.76 and 2.71. The number of rotatable bonds (nRTB) for 8 phytoconstituents, DB09, DB12, DB16, DB19, DB28, DB63, DB64, and DB66, were within the recommended range of ≤10. It is also evident from Table 2 that values of the topological surface area (TPSA) were relatively high except for DB09, DB12, and DB19 which were not more than 140 Å^2^. The result presented in Table 2 also shows that 5 compounds, DB09, DB12, DB19, DB28, and DB66, followed the Ro5, and only 3 compounds, DB9, DB12, and DB19, did not violate Veber's rule.

The pharmacokinetic profiling of the selected phytoconstituents is presented in Table 3. The calculated parameters revealed that only DB09, DB12, and DB19 possess high intestinal absorption, and five of the compounds (DB05, DB06, DB07, DB10, and DB63) are P-glycoprotein (P-gp) substrates. Similarly, the predicted oral bioavailability (F_20%_+) ranged from 0.01 to 0.97. Distribution-related parameters as presented in Table 3 also indicated that the plasma binding protein (PPB) of the phytoconstituents is in the range of 20.90 to 98.92%. The prediction for the blood-brain barrier (BBB) penetration returned “No” for all the compounds, while the fraction unbound in plasma (FU) showed that all the compounds except DB09 and DB12 are within the recommended range of ≥5. Similarly, the values for the volume of distribution (VD) revealed that all the phytoconstituents were found within the range of 0.04-20. The metabolism with respect to CYP450 3A4 and CYP450 2D6 isozymes was also predicted. The analysis revealed that all the compounds except for DB19 are not likely to inhibit any of the isozymes (Table 3). For excretion, the predicted total clearance was found in a range of 1.32 to 16.93, existing within the recommended range of ≥5.

As shown in Table 4, toxicity predicted by ADMETlab revealed that the compounds are free of human Ether-à-go-go-Related Gene (hERG), and human hepatotoxicity effects, as values for these properties were within the recommended safe range of 0-0.3. Similarly, toxicity as calculated by Osiris property explorer also indicated that the hit compounds might not induce mutagenicity, tumorigenicity, and reproductive effects except for DB07, DB19, and DB64 which were predicted as highly mutagenic and tumorigenic.

### 3.3. Molecular Dynamics Simulation

The binding stability and information on intermolecular interactions of docking complexes within a reference time can be investigated using MD simulation [47]. The current study analyzed the conformational stability and interactions of the protein-ligand docked complexes of KPC-2 protein and selected hit compounds (DB09, DB12, DB28, and DB66) over a 150 ns time interval using their docking files. The results of these analyses are presented as follows.

#### 3.3.1. Analyses of the RMSD, RMSF, rGyr, and SASA Properties of the Complexes

**Figure 4:**
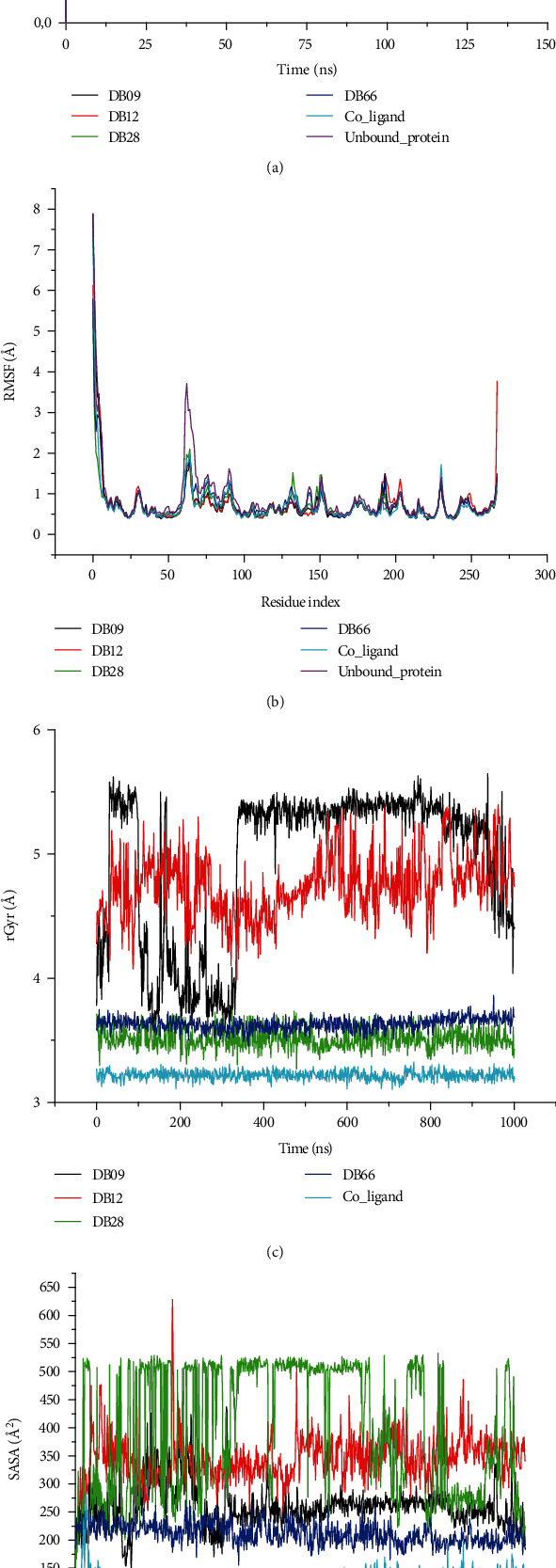
The MD simulation of the KPC-2 protein and selected hit compounds (DB09, DB12, DB28, and DB66) and coligand throughout the 150 ns simulations. (a–d) The analyses of RMSD, RMSF, rGyr, and SASA for the five complexes, respectively.

As shown in Figure 4(a), the C*α* atoms of KPC-2 protein in complex with DB09, DB12, DB28, and DB66 have an average RMSD value of 1.324 ± 0.16, 1.398 ± 0.18, 1.207 ± 0.19, and 1.349 ± 0.22 Ǻ, respectively (Table S2). An average RMSD value of 1.200 ± 0.31 was also observed for the coligand while the unbound KPC-2 protein had an average value of 1.456 ± 0.38 throughout the entire simulation period.

Next, the deviations generated by the residue index were used to determine the local structural fluctuations (P-RMSF) of the C*α* atoms of the KPC-2 protein only, and when in complex with the compounds. The peaks in Figure 4(b) indicate the areas concerning the residue index of the protein that was perturbed during the 150 ns simulation period. The N- and C-terminal residues for all the complexes fluctuated significantly more than any other parts of the protein, reaching a peak of above 3.0 Å. All other secondary structures remained consistent during the trajectories keeping an average RMSF below 0.9 Å to demonstrate conformational stability (Table S2). The RMSF of the protein complex with the coligand was also steady and plummeted.

The residual compactness of the complexes was explored by determining the radius of gyration from the MD simulation trajectories. The values of rGyr plotted against the simulation time are presented in Figure 4(c), and the average values are given in Table S2. These values were found to be 4.944 ± 0.62, 4.745 ± 0.25, 3.514 ± 0.08, 3.631 ± 0.05, and 3.219 ± 0.04 Ǻ for the complexes produced by DB09, DB12, DB28, DB66, and the coligand, respectively. Similarly, the SASA of the simulated complexes was also analyzed to understand the surface area of the molecule that is accessible to the solvent. The SASA profiles are presented in Figure 4(d), and the complexes of DB09, DB12, DB28, DB66, and coligand had an average SASA value of 259.92, 345.99, 401.67, 211.89, and 121.01 Ǻ^2^, respectively.

#### 3.3.2. Binding Interaction Analyses

**Figure 5:**
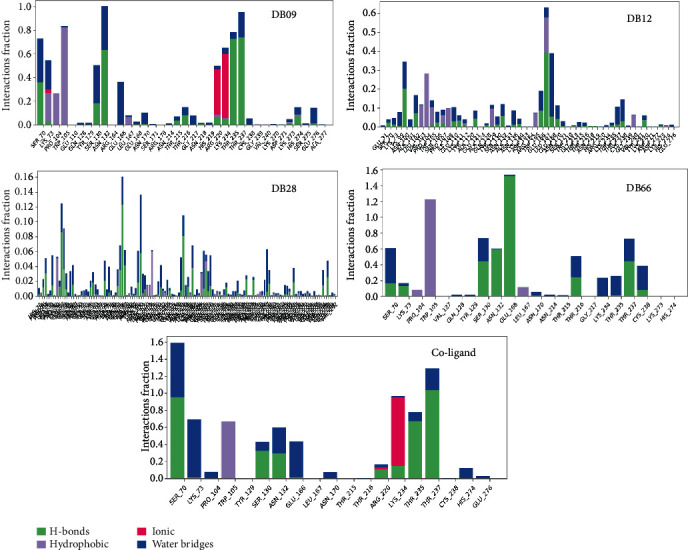
KPC-2 protein interactions with selected *Ehretia* species phytoconstituents throughout the 150 ns MD simulations. The interactions include hydrogen bonds, water bridges, ionic, and hydrophobic bonds.

The MD simulations were further analyzed to find out a change in the interaction behaviour of the docked complexes. The possible interactions, such as hydrogen bonds, water bridges, and ionic, and hydrophobic interactions were monitored for the five complexes during the entire simulations (Figure 5). Hydrogen bonds play a key role in ligand binding, and the dynamic equilibration of the protein-ligand complex trajectories revealed the distribution of hydrogen bond interactions across various complexes (Figure S1). Furthermore, protein-ligand contacts as described in Figure 5 showed that Ser70, Lys73, Ser130, Glu166, Thr235, and Thr237 predominantly stabilize the complexes using hydrogen bonds. Similarly, Trp105 and Lys234 showed significant hydrophobic and ionic interactions, respectively, while Lys73 of the protein targets predominantly formed water bridge interaction with the ligands. The stability of these interactions as described in Figure S2 showed a range between 30 and 98% of the full-scale time trajectories. However, all the interactions formed by DB28 were lost before 30% of the simulation time.

#### 3.3.3. Comparison of the Docked Complexes before and after MD Simulations

**Figure 6:**
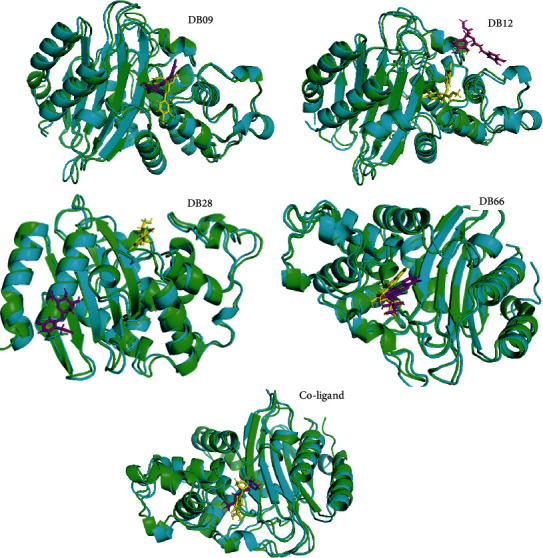
The conformations of the ligands within the active site of KPC-2 protein before (green chain) and after (blue chain) MD simulation. The conformations of each ligand before and after the simulation are represented as yellow and magenta colour, respectively.

Superimposition of the docked complexes and the last snapshot from the MD simulation trajectory was performed to determine a change in the conformational behaviour of the docked complexes. The results as presented in Figure 6 established that both structures had a similar binding conformation in the active site of the KPC-2 protein in complex with DB66 and the coligand. However, DB12 and DB28 experienced more significant conformational shifts during the MD trajectory. This agreed with the above binding analysis since DB28 left the binding pocket and became solvent-exposed during the MD simulation run.

## 4. Discussion

Computational or *in silico* approaches are becoming increasingly important in contemporary drug discovery initiatives, as they are vital in the early identification of viable drug candidates often at a faster pace and lower cost than traditional methods. These approaches also reduce the use of animal models in pharmacological research, assisting in the rational design of novel and safe drug candidates, repurposing existing therapeutic agents, as well as helping medicinal chemists and pharmacologists throughout the drug discovery process [53, 54]. KPC-2 is a class A active site serine beta-lactamase that hydrolyzes beta-lactams through a covalent acyl-enzyme [12]. Almost all beta-lactam antibiotics, including carbapenems and most cephalosporins, can be hydrolyzed by KPC-2. It is a prominent cause of carbapenem and other beta-lactam failures in healthcare-associated infections. This is due to the widespread endemic presence and their hydrolytic activity against broad-spectrum beta-lactams [12, 55, 56]. Unfortunately, there is no “ideal” treatment for infections caused by KPC-2-expressing bacteria at present, and data on clinical outcomes are limited due to the therapeutically decreasing options [55, 57]. That said, the current study used *in silico* approaches to evaluate the efficacy of *Ehretia* species phytoconstituents as potential inhibitors of the KPC-2 protein to reduce the burden of infections caused by KPC-expressing bacteria.

Before the docking studies, the protocol was validated as described in the methodology section. The RMSD of the coligand and after it was redocked was used to assess the reproducibility of the docking algorithm. Previous studies have reported that an RMSD value ≤ 2 Å is satisfactory for validating docking experiments [58, 59]. Therefore, a good and reliable process was used for the docking of the *Ehretia* species phytoconstituents to the active sites of the KPC-2 protein target in the present study.

The top-ranked compounds obtained from the docking studies are predominantly phenolic acids and flavonoids. Khanh and Hoa [35] recently conducted a virtual screening of selected medicinal plants against protease enzyme in COVID-19 and identified 15 hits which were majorly flavonoids and phenolic acids. Kępa et al. [60] also reported the inhibitory potential of phenolic acids (such as caffeic acid) against a wide range of bacteria including *S. aureus*, *K. pneumoniae*, *P. mirabilis*, *E. coli*, and *P. aeruginosa*. Furthermore, flavonoids (e.g., quercetin, hyperoside, and rutin) have shown strong inhibitory properties against *P. aeruginosa*, *Klebsiella* species, and other Enterobacteriaceae strains [28, 61, 62]. Some of these phytoconstituents have also been reported to have synergistic activity in combination with antibiotics against *β*-lactamase expressing *K. pneumoniae* [60, 63]. Therefore, the observed binding affinities of the top-ranked phytoconstituents in this study might indicate their efficacy against the KPC-2 enzyme.

The residues Ser70, Trp105, Ser130, Asn132, Asn235, and Thr237 were predominantly involved in the binding interactions of most of the compounds within the active site of the KPC-2 protein. Previous studies have shown that Ser130, Lys234, Thr235, and Thr237 are conserved residues in the active site of KPC-2 protein. The role of Trp105 in substrate and inhibitor interactions leading to hydrolysis in KPC-2-lactamase has also been reported [56, 64–66]. Furthermore, a study by Papp-Wallace et al. [67] has shown that Ser130 is actively involved in the mechanism of KPC-2 resistance to avibactam. Therefore, the predicted binding interactions in this study might imply strong biological activities of the *Ehretia* species phytoconstituents.

The MM-GBSA calculation is a postdocking approach that is extensively used to predict an end-point binding free energy and with the benefit of producing more reliable results than most molecular docking functions [68]. A more negative binding free energy (dG bind) of protein-ligand complexes indicates better stability of the interaction [69]. The predicted binding free energy in this study revealed that the *Ehretia* species phytoconstituents established stronger and more favorable binding with the KPC-2 protein than the coligand.

The physicochemical, drug-likeness, pharmacokinetics, and toxicity properties of the hit phytoconstituents were profiled to identify the extent of their similarity to the known drugs. The most often studied physicochemical parameters in drug discovery research include MW, LogS, HBA, HBD, nRTB, and LogP [70, 71]. These parameters have been extensively investigated for their ability to influence several pharmacokinetic aspects such as absorption, bioavailability, permeability, and elimination, particularly in the case of oral drugs [33, 72]. It is apparent from this study that only DB09, DB12, and DB19 showed prospects for good oral bioavailability and penetration through biological membranes. This is because they exist within the recommended limits of 500 for MW, ≤10 for HBA, ≤5 for HBDs, and ≤140 for TPSA. Many naturally derived antibacterial agents, including azithromycin, paclitaxel, and streptogramins, exist outside these limits. In addition, larger molecules tend to encumber the binding pockets of therapeutic targets to generate bioactivity [33, 73, 74]. This could explain why some of the top-ranked *Ehretia* species phytoconstituents had higher MWs.

Log*P* is an essential parameter that connects solubility, membrane permeability, drug absorption, and distribution with metabolic or renal clearance [75]. An increase in Log*P* might influence the interaction between the hydrophobic region of the drug and its targets, thereby increasing the drug's efficacy. In addition, a negative Log*P* value shows that a molecule is hydrophobic, while a positive value indicates that the molecule might have a higher affinity for the lipophilic phase. However, a molecule with a Log*P* value above 3 might be an indication of drug promiscuity and toxicity [33, 74]. Similarly, the Log*S* of a compound has a significant impact on the absorption and distribution rates. Low-soluble drugs are most likely to be poorly absorbed, and the main goal of drug development is to avoid poorly soluble compounds [48, 76]. Interestingly, the observed values for both Log*P* and Log*S* in the present study imply that the top-ranked ligands might have good oral bioavailability and permeability.

Drug-likeness is a quantitative concept of drug design that describes compounds with functional groups and chemical and physical properties that are similar to those of the existing drugs [77]. The recorded findings from this study show that only DB09, DB12, DB19, DB28, and DB66 obeyed the Ro5 while DB28 and DB66 additionally followed Veber's rule. The Ro5 describes the ranges at which a set of four physicochemical parameters would increase the likelihood of oral bioavailability and good pharmacokinetic profile in drug design. The rule states that drug-like molecules may not violate more than one of the following conditions: MW ≤ 500 g/mol, Log*P* ≤ 5, HBA ≤ 5, and HBD ≤ 5 to exhibit good membrane permeability [33, 78, 79]. Similarly, Veber's rules state that molecules with 10 or fewer nRTB and TPSA equal to or lesser than 140 Å2 are likely to have good oral bioavailability [31, 80].

The early determination of the pharmacokinetic and toxicological properties of drug candidates remains a critical step in drug development [81]. We observed that only DB09, DB12, and DB19 showed high propensities for passive absorption. The predicted range of values for PPB, FU, and VD indicated that most of the hit phytoconstituents are within the space of known drug molecules, and the limiting effect of their distribution by the BBB might be beneficial to their safety profiles. It was also established that only DB19 might be a substrate for cytochrome P450 isozymes, indicating the potential for toxic drug accumulation and clinically significant drug-drug interactions. The results for the toxicity profile implied that all *Ehretia* species phytoconstituents were not likely to be toxic to hERG and with negligible or no human hepatotoxicity effects. Similarly, based on the results of Osiris property explorer, DB07 was found to be tumorigenic while DB19 and DB64 were shown to have mutagenic and tumorigenic effects.

The physicochemical, pharmacokinetic, and toxicological profiling of the phytoconstituents revealed that DB09, DB12, DB28, and DB66 could be orally administered drug candidates. The binding energy of these compounds shows that they assumed good orientation through different interactions. Therefore, MD simulation was performed to gain insight into protein conformation, and stability of the complexes formed by these phytoconstituents with the KPC-2 protein. The magnitude of deviations as shown by RMSD indicates that all the systems attained stable conformations after equilibration. The RMSF of the complexes also remained steady and comparable with the unbound protein reporting no significant fluctuations. However, the high average rGyr values observed for the phytoconstituents, particularly DB09 and DB12 in comparison with the coligand, indicate less compactness and folding behaviour of the complexes. This could be attributed to conformational variations affecting the secondary structure of the protein during the MD simulations [82]. The complexes of DB09, DB66, and coligand demonstrate lesser SASA amplitude, indicating more compactness while the higher SASA amplitude observed for DB12 and DB28 indicated an extended surface volume which can result in less structural stability. Furthermore, intermolecular hydrogen bond assessment also agrees with the result of other descriptors from MD simulation as they were moderately stable across the simulation trajectory. Finally, the drastic changes in the binding poses of these ligands, particularly DB12 and DB28, indicated that the predicted protein-ligand interactions involving these phytoconstituents are not reliable.

## 5. Conclusions

The present study explored and demonstrated that, among the 69 *Ehretia* species phytoconstituents, 14 exhibited a more promising binding affinity for the KPC-2 protein target than the reference drugs. These compounds predominantly belong to the class of phenolic acids and flavonoids. Only 4 of these phytoconstituents, DB09, DB12, DB28, and DB66, are drug-like and present no significant toxicity. In addition, MD simulations have profiled the stability of these compounds and the potential conformational changes that they can incite on the protein throughout a 150 ns run. We also established that the catalytic residue of the KPC-2, Ser130, Lys234, Thr235, Thr237, and Trp105 binds with the hit compounds, and these were also sustained in the post-MD structures of most of the complexes. The therapeutic potential of natural compounds, as evaluated in this study, could be of additional benefit in the search for novel drug candidates against KPC-2-expressing bacterial strains. Future studies will futher investigate the stability of the respective complex and validate the ability of these compounds, particularly DB66, to inhibit the KPC-2 protein target.

## Figures and Tables

**Table 1:** Binding affinities and intermolecular bonds of the top hit *Ehretia* species phytoconstituents, and the coligand within the KPC-2 protein active site.

ID	Compound	Phytoconstituent group	Docking score (kcal/mol)	MM-GBSA dG bind ((kcal/mol))	No. of H-bonds and the participating residues	Other bonds and the participating residues
DB01	Buddlenol B	Phenolic	-8.89	-64.42	5 (Ser70, Asn132, Asn170, Thr215, Thr216)	*π*-*π* stacking (Trp105)
DB05	Ehletianol A	Phenolic	-8.60	-54.10	7 (Asn132, Thr215, Thr235, Thr237, Hie274, Glu276^2^)	—
DB06	Ehletianol B	Phenolic	-8.66	-53.48	7 (Asn132^2^, Asn218, Thr237^2^, Hie274, Glu276)	*π*-*π* stacking (His219)
DB07	Ehletianol D	Phenolic	-10.42	-47.35	6 (Ser70, Ser130^2^, Thr235, Thr237^2^)	*π*-*π* cation (Trp105)
DB09	Caffeic anhydride	Phenolic	-7.47	-46.25	5 (Ser130, Asn170^2^, Thr235, Thr237)	Salt bridge (Lys234)
DB10	Icariside E5	Phenolic	-11.13	-60.39	7 (Ser70^2^, Asn132, Asn170^2^, Hie274, Glu276)	—
DB11	Lithospermic acid B	Phenolic	-10.95	-79.36	4 (Ser70, Thr216, Thr235, Thr237)	*π*-*π* stacking (Trp105)
DB12	Methyl rosmarinate	Phenolic	-8.98	-53.20	5 (Ser70^2^, Asn170^2^, Thr235)	—
DB16	Hyperoside	Flavonoid	-10.71	-62.84	7 (Ser70, Ser130^2^, Asn132^2^, Asn170, Thr237)	*π*-*π* stacking (Trp105^3^)
DB19	Quercetin	Flavonoid	-7.53	-44.79	3 (Ser70, Thr216^2^)	*π*-*π* stacking (Trp105)
DB28	Ehretioside B	Glycoside	-8.79	-48.91	3 (Asn132, Asn170, Thr237)	*π*-cation (Lys234)
DB63	Rutin	Flavonoid	-12.55	-66.33	7 (Ser70^2^, Asn132, Leu167, Thr235, Thr237, Cys238)	*π*-*π* stacking (Trp105^3^)
DB64	Lucenin 2	Flavonoid	-11.23	-58.65	5 (Ser70, Asn132^2^, Thr237^2^)	—
DB66	Riboflavin	Vitamin	-9.61	-64.63	5 (Ser70, Asn170, Thr235, Thr237, Cys238)	—
Coligand	Faropenem	Reference	-7.33	-14.30	6 (Ser70, Ser130, Asn132, Thr235, Thr237^2^)	Salt bridge (Lys73, Lys234); *π*-cation (Lys73)

Amino acid residues with numbers (n (a)) represent the total residues involved in the interaction while amino acid residues with superscripts (a^n^) represent the number of bonds participating in the interaction.

**Table 2:** Physicochemical and drug-likeness properties of the *Ehretia* species phytoconstituents.

ID	Ligands	MW (g/mol)	Log*S*	Log*P*	HBA	HBD	nRTB	TPSA	Ro5 violations	Veber's violations
Requirements		≤500	-4 ~ 0.5	≤5	≤10	≤5	≤10	≤140 Å2	<2	0
DB01	Buddlenol B	584.61	-3.739	1.692	11	5	13	156.53	2	2
DB05	Ehletianol A	794.71	-3.007	0.752	20	8	16	288.28	3	2
DB06	Ehletianol B	764.68	-3.116	0.797	19	8	15	279.05	3	2
DB07	Ehletianol D	520.53	-2.687	-0.131	11	6	11	175.37	3	2
DB09	Caffeic anhydride	342.30	-2.588	2.71	7	4	6	124.29	0	0
DB10	Icariside E5	522.54	-2.135	-0.444	11	7	11	178.53	3	2
DB11	Lithospermic acid B	718.61	-3.88	2.216	16	9	14	278.04	3	2
DB12	Methyl rosmarinate	374.34	-2.337	2.197	8	4	8	133.52	0	0
DB16	Hyperoside	464.38	-3.871	-0.17	12	8	4	210.51	2	1
DB19	Quercetin	302.24	-3.671	2.155	7	5	1	131.36	0	0
DB28	Ehretioside B	311.29	-1.254	-1.084	8	5	4	143.40	0	1
DB63	Rutin	610.52	-3.93	-0.76	16	10	6	269.43	3	2
DB64	Lucenin 2	610.52	-2.41	-1.36	16	12	5	291.43	3	2
DB66	Riboflavin	376.36	-3.657	-0.438	8	5	5	161.56	0	1

MW = molecular weight; Log*S* = logarithm of aqueous solubility value; Log*P* = octanol-water partition coefficient; HBA = hydrogen bond acceptor; HBD = hydrogen bond donor; nRTB = number of rotatable bonds; TPSA = topological surface area; Ro5 violation = violations from the rule of five.

**Table 3:** *In silico* pharmacokinetic properties for the *Ehretia* species phytoconstituents.

ID		Absorption			Distribution				Metabolism		Excretion
		GI absorption	P-gp substrate	F_20%_	PPB (%)	BBB permeate	FU (%)	VDSS	CYP3A4 inhibitor	CYP2D6 inhibitor	Total clearance
Requirements	Ligands	High	No	0 ~ 0.7	≤90	No	≥5	0.04~20	No	No	≥5
DB01	Buddlenol B	Low	No	0.01	80.08	No	19.81	0.80	No	No	6.90
DB05	Ehletianol A	Low	Yes	0.04	64.33	No	35.88	0.48	No	No	6.60
DB06	Ehletianol B	Low	Yes	0.05	72.79	No	22.19	0.52	No	No	6.89
DB07	Ehletianol D	Low	Yes	0.15	88.17	No	8.80	0.57	No	No	4.59
DB09	Caffeic anhydride	High	No	0.97	98.92	No	1.20	0.40	No	No	14.31
DB10	Icariside E5	Low	Yes	0.32	72.53	No	15.54	0.59	No	No	4.46
DB11	Lithospermic acid B	Low	No	0.97	95.52	No	5.17	0.34	No	No	9.22
DB12	Methyl rosmarinate	High	No	0.79	95.57	No	3.22	0.42	No	No	16.93
DB16	Hyperoside	Low	No	0.48	86.41	No	15.12	0.90	No	No	5.37
DB19	Quercetin	High	No	0.93	95.49	No	7.42	0.58	Yes	Yes	8.28
DB28	Ehretioside B	Low	No	0.15	20.90	No	69.25	0.58	No	No	2.60
DB63	Rutin	Low	Yes	0.23	83.81	No	20.86	0.75	No	No	1.35
DB64	Lucenin 2	Low	No	0.97	76.79	No	17.82	0.91	No	No	1.32
DB66	Riboflavin	Low	No	0.02	81.58	No	21.69	0.61	No	No	5.67

GI = gastrointestinal tract; P-gp = P-glycoprotein; F_20%_ = human oral bioavailability; PPB = plasma protein binding; FU (%) = fraction unbound in plasma; VD = volume distribution; BBB = blood-brain barrier; CYP = cytochrome P450.

**Table 4:** *In silico* toxicological properties of the *Ehretia* species phytoconstituents.

ID	Ligands	hERG blocker	Mutagenicity	Tumorigenicity	Reproductive effect	Human hepatotoxicity
Requirements		0 ~ 0.3				0 ~ 0.3
DB01	Buddlenol B	0.15	None	None	None	0.293
DB05	Ehletianol A	0.101	None	None	None	0.016
DB06	Ehletianol B	0.062	None	None	None	0.011
DB07	Ehletianol D	0.104	Low	High	None	0.318
DB09	Caffeic anhydride	0.007	None	None	None	0.242
DB10	Icariside E5	0.071	None	None	None	0.404
DB11	Lithospermic acid B	0.025	None	None	None	0.381
DB12	Methyl rosmarinate	0.032	None	None	None	0.227
DB16	Hyperoside	0.017	None	None	None	0.144
DB19	Quercetin	0.099	High	High	None	0.1
DB28	Ehretioside B	0.009	None	None	None	0.082
DB63	Rutin	0.017	None	None	None	0.092
DB64	Lucenin 2	0.035	High	High	Low	0.147
DB66	Riboflavin	0.029	None	None	None	0.118

hERG: human Ether-à-go-go-Related Gene.

## Data Availability

All data can be found within the manuscript and the supplemental materials.

## Abbreviations

PDB: Protein Data Bank
KPC: *Klebsiella pneumoniae* carbapenemase
MD: Molecular dynamics
AMR: Antimicrobial resistance
SMILES: Simplified molecular-input line-entry system
RMSD: Root-mean-square deviation
XP: Extra precision
ADMET: Absorption, distribution, metabolism, excretion, and toxicity
Ro5: Rule of five
TIP-3P: Three-site transferrable intermolecular potential
RMSF: Root-mean-square fluctuation
HBA: Hydrogen bond acceptors
HBD: Hydrogen bond donors
MW: Molecular weight
TPS: Topological surface area
LogS: Logarithm of aqueous solubility
LogP: Octanol-water partition coefficient
nRTB: Number of rotatable bonds
P-gp: P-glycoprotein
BBB: Blood-brain barrier
FU: Fraction unbound in plasma
VD: Volume of distribution
CYP: Cytochrome P450
GI: Gastrointestinal tract
P-gp: P-glycoprotein
PPB: Plasma protein binding
hERG: Ether-à-go-go-related gene
ROA: Rat oral acute toxicity.

## Supplementary Materials

Supplementary Materials Supplementary Table S1: a library of *Ehretia* species phytoconstituents obtained from the literature. Supplementary Table S2: the RMSD, RMSF, and rGyr values of the complexes are expressed as Mean ± SD (Å). Figure S1: post-MD analysis plots representing the number of interacting hydrogen bonds of the protein-ligand complex trajectories.
