# Supplementary material for: Ehretia Species Phytoconstituents as Potential Lead Compounds against Klebsiella pneumoniae Carbapenemase: A Computational Approach
Source: Biomed Res Int. 2023 Oct 12;2023:8022356. doi: 10.1155/2023/8022356 (PMC10586912; doi:10.1155/2023/8022356)
Supplement: Supplementary Materials — Supplementary Table S1: a library of Ehretia species phytoconstituents obtained from the literature. Supplementary Table S2: the RMSD, RMSF, and rGyr values of the complexes are expressed as Mean ± SD (Å). Figure S1: post-MD analysis plots representing the number of interacting hydrogen bonds of the protein-ligand complex trajectories. [file 8022356.f1.docx]

***Ehretia* species phytoconstituents as potential lead compounds against *Klebsiella pneumoniae* carbapenemase *-* A computational approach.**

Samson O Oselusi^1,2^, Nicole RS Sibuyi^2^, Mervin Meyer^2^, and Abram M Madiehe^1,2^*

Samson O Oselusi: 3866891@myuwc.ac.za

 Nicole RS Sibuyi: nsibuyi@uwc.ac.za

Mervin Meyer: memeyer@uwc.ac.za

Abram M Madiehe: amadiehe@uwc.ac.za

*Corresponding author: Abram M Madiehe, amadiehe@uwc.ac.za

Table S1. Presents a library of *Ehretia species* phytoconstituents obtained from the literature.

| ID | Phytoconstituent | Class of phytoconstituent | Docking score | Glide energy |
| --- | --- | --- | --- | --- |
| DB01 | Buddlenol B | Phenolic | -8.600 | -55.713 |
| DB02 | Caffeic acid | Phenolic | -6.948 | -33.818 |
| DB03 | Danshensu | Phenolic | -7.153 | -35.579 |
| DB04 | Cinnamic acid | Phenolic | -3.619 | -23.717 |
| DB05 | Ehletianol A | Phenolic | -8.787 | -58.219 |
| DB06 | Ehletianol B | Phenolic | -8.968 | -61.854 |
| DB07 | Ehletianol D | Phenolic | -10.708 | -51.299 |
| DB08 | (E)-ethyl caffeate | Phenolic | -5.272 | -32.619 |
| DB09 | Caffeic anhydride | Phenolic | -7.467 | -47.250 |
| DB10 | Icariside E5 | Phenolic | -10.950 | -55.867 |
| DB11 | Lithospermic acid B | Phenolic | -10.424 | -69.321 |
| DB12 | Methyl rosmarinate | Phenolic | -8.894 | -44.387 |
| DB13 | Rosmarinic acid | Phenolic | -7.138 | -53.867 |
| DB14 | Ehletianol C | Phenolic | -5.530 | -53.144 |
| DB15 | Apigenin | Flavonoid | -6.580 | -37.110 |
| DB16 | Hyperoside | Flavonoid | -11.127 | -56.385 |
| DB17 | Luteolin | Flavonoid | -7.007 | -43.466 |
| DB18 | Kaempferol | Flavonoid | -5.658 | -34.513 |
| DB19 | Quercetin | Flavonoid | -7.526 | -36.877 |
| DB20 | Ovalifolin | Flavonoid | -6.236 | -36.146 |
| DB21 | Ehretinine | Alkaloid | -3.545 | -30.930 |
| DB22 | Allantoin | Alkaloid | -5.817 | -31.739 |
| DB23 | Araneosol | Fatty acid | -6.241 | -39.425 |
| DB24 | Ehretianone | Benzoquinone | -3.566 | -30.197 |
| DB25 | Microphyllone | Benzoquinone | -4.322 | -27.483 |
| DB26 | Ehretiquinone | Benzoquinone | -4.153 | -29.911 |
| DB28 | Ehretioside B | Glycoside | -8.658 | -42.309 |
| DB29 | Bauerenol | Other | -3.974 | -35.961 |
| DB30 | Bauerenol acetate | Other | -2.982 | -33.531 |
| DB31 | Betulinic acid | Other | 0.893 | -33.908 |
| DB32 | Lupeol | Other | -2.552 | -29.878 |
| DB33 | β-sitosterol | Other | -3.659 | -31.900 |
| DB34 | Daucosterol | Other | -3.889 | -29.333 |
| DB35 | Stigmasterol | Other | -5.510 | -30.422 |
| DB36 | α-spinasterol | Other | -5.417 | -42.169 |
| DB37 | Campesterol | Other | -2.323 | -20.202 |
| DB38 | Stigmastanol | Other | -2.613 | -22.633 |
| DB39 | Ehretiolide | Other | -4.917 | -36.761 |
| DB40 | Ehreticoumarin | Other | -4.682 | -28.958 |
| DB41 | Ehretilactone A | Other | -4.065 | -23.278 |
| DB42 | Ehretilactone B | Other | -5.840 | -25.946 |
| DB43 | Ehretiamide | Other | -5.502 | -29.307 |
| DB44 | Ehretiate | Other | -2.030 | -42.899 |
| DB45 | Linalool | Essential oil | -3.764 | -23.734 |
| DB46 | Trans-Sesquisabinene hydrate | Essential oil | -4.611 | -26.349 |
| DB47 | Methyl salicylate | Essential oil | -4.374 | -24.695 |
| DB48 | β-Cedrene | Essential oil | -3.130 | -21.773 |
| DB49 | β-Damascenone | Essential oil | -3.720 | -23.383 |
| DB50 | β-Ylangene | Essential oil | -2.731 | -17.046 |
| DB51 | 1,4-naphthoquinone | Essential oil | -4.339 | -23.646 |
| DB53 | ar-Curcumene | Essential oil | -2.317 | -21.243 |
| DB54 | β-Bisabolene | Essential oil | -1.462 | -21.491 |
| DB55 | Lupane | Pentacyclic Triterpenoid | -1.788 | -22.512 |
| DB56 | Ursane | Pentacyclic Triterpenoid | -2.705 | -25.782 |
| DB57 | Betulin | Pentacyclic Triterpenoid | -3.350 | -27.828 |
| DB58 | Ursolic acid | Pentacyclic Triterpenoid | -3.477 | -34.313 |
| DB59 | Hesperetin | Flavonoid | -6.512 | -40.737 |
| DB60 | Naringenin | Flavonoid | -6.897 | -41.532 |
| DB61 | Catechin | Flavonoid | -6.695 | -43.743 |
| DB62 | Genistein | Flavonoid | -5.084 | -43.034 |
| DB63 | Rutin | Flavonoid | -12.554 | -60.902 |
| DB64 | Lucenin 2 | Flavonoid | -11.223 | -48.011 |
| DB65 | Hematoporphyrin | Other | -4.356 | -47.670 |
| DB66 | Riboflavin | Vitamin | -8.976 | -50.043 |
| DB67 | Alpha-Tocopherol | Vitamin | -3.875 | -34.996 |
| DB68 | Retinol | Vitamin | -5.064 | -29.313 |
| DB69 | Thiamine | Vitamin | -4.266 | -35.761 |
| DB70 | Relebactam | Control | -4.307 | -40.746 |
| DB71 | Avibactam | Control | -4.842 | -36.727 |
| DB72 | Vaborbactam | Control | -5.671 | -31.929 |
| Co-ligand | Faropenem | Control | -7.333 | -40.037 |

Table S2: The RMSD, RMSF and rGyr values of the complexes expressed as Mean ± SD (Å), and SASA Mean ± SD (Å^2^).

| Complex | RMSD | RMSF | rGyr | SASA |
| --- | --- | --- | --- | --- |
| KPC-2_DB09 | 1.324 ± 0.16 | 0.734 ± 0.67 | 4.944 ± 0.62 | 259.92 ± 37.46 |
| KPC-2_DB12 | 1.398 ± 0.18 | 0.757 ± 0.59 | 4.745 ± 0.25 | 345.99 ± 40.63 |
| KPC-2_DB28 | 1.207 ± 0.19 | 0.725 ± 0.43 | 3.514 ± 0.08 | 401.67 ± 112.71 |
| KPC-2_DB66 | 1.349 ± 0.22 | 0.760 ± 0.51 | 3.631 ± 0.05 | 211.89 ± 18.79 |
| KPC-2_coligand | 1.200 ± 0.31 | 0.731 ± 0.59 | 3.219 ± 0.04 | 121.01 ± 21.27 |
| Unbound protein | 1.456 ± 0.39 | 0.888 ± 0.77 | NA | NA |


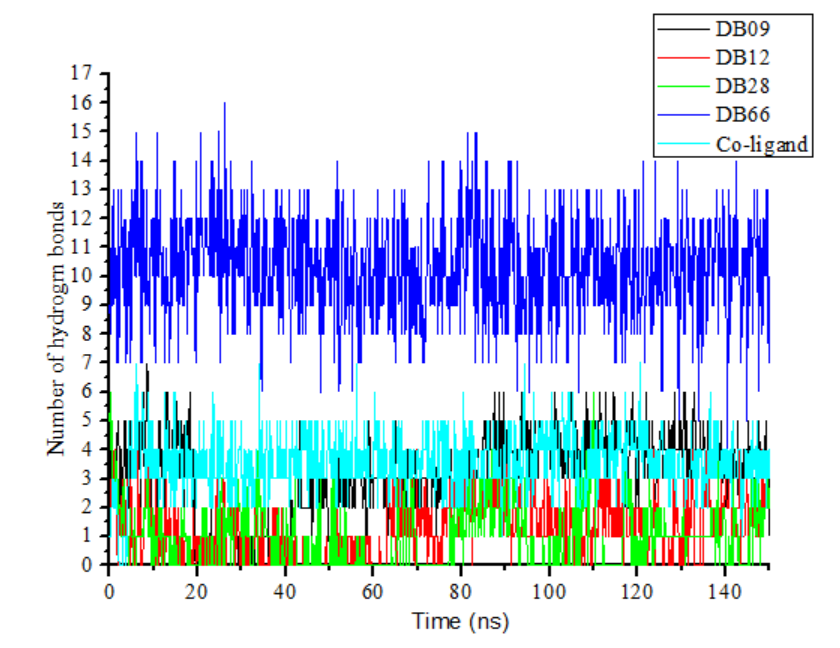


Figure S1: Post MD analysis plots representing the number of interacting hydrogen bonds of the protein-ligand complexes trajectories.

| 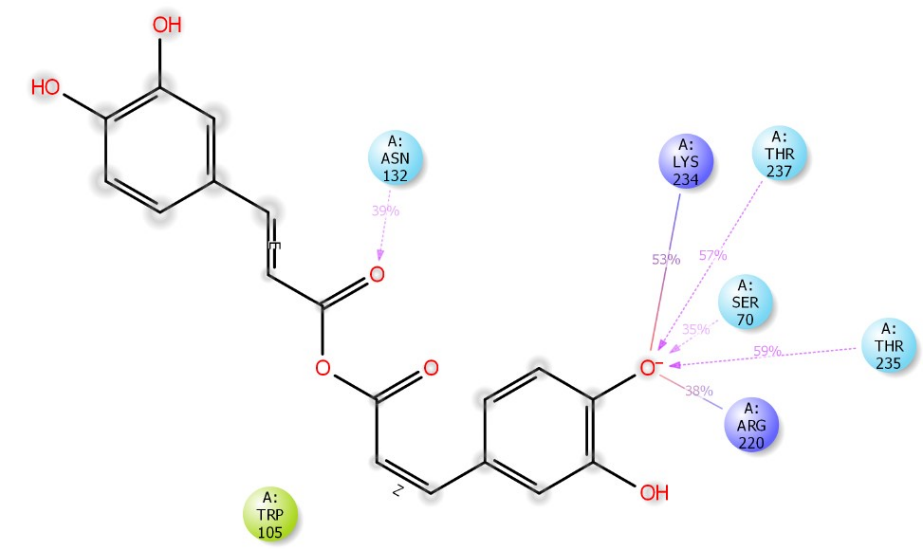  DB09 | DB12  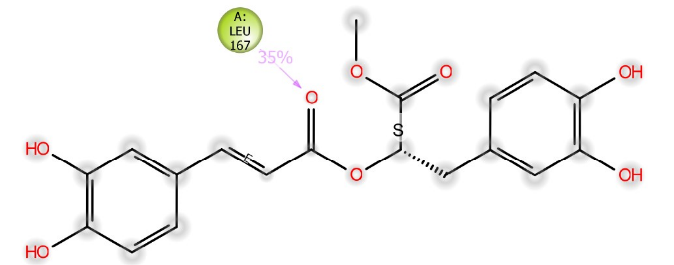 |
| --- | --- |
| 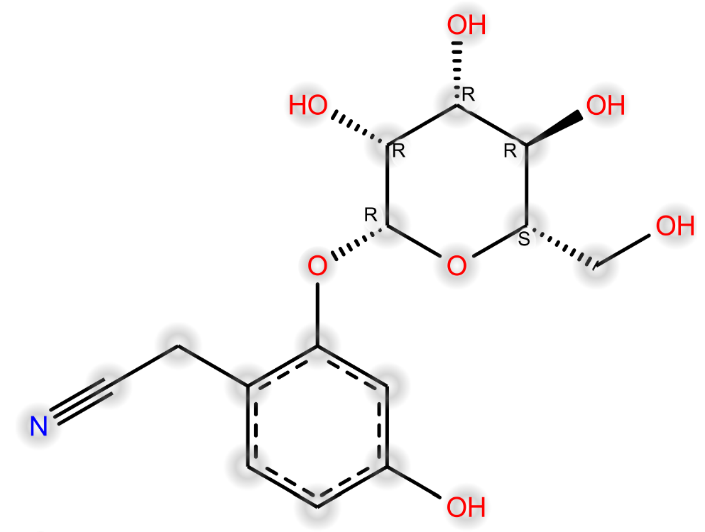  DB28 | 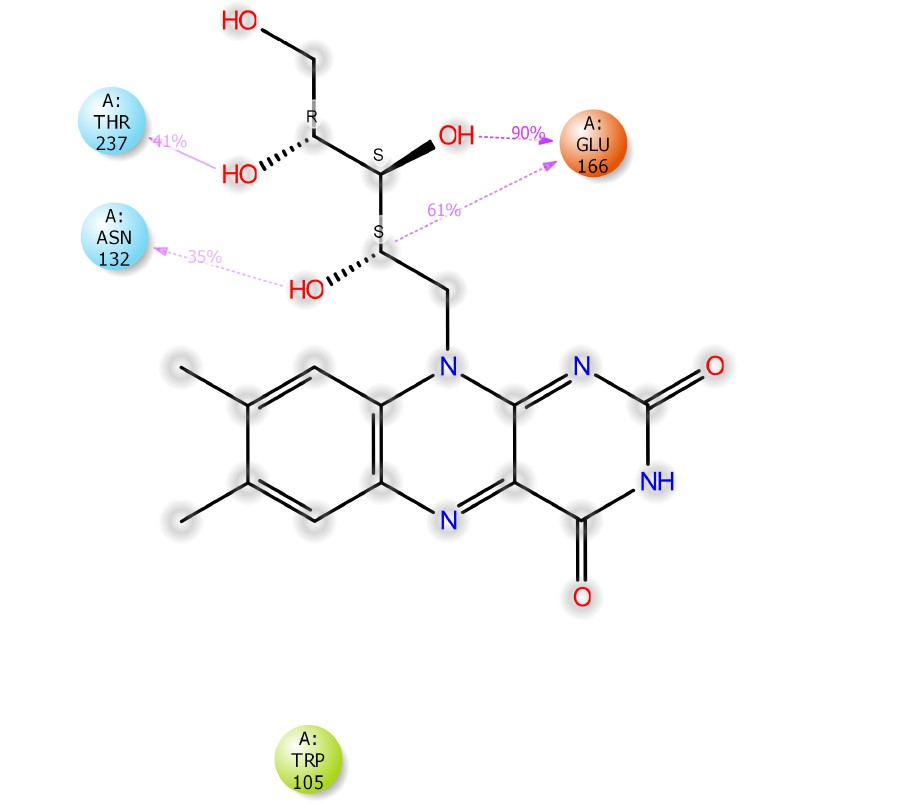  DB66 |
| 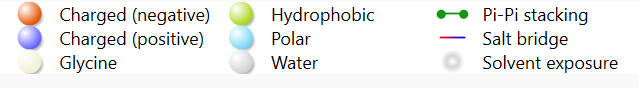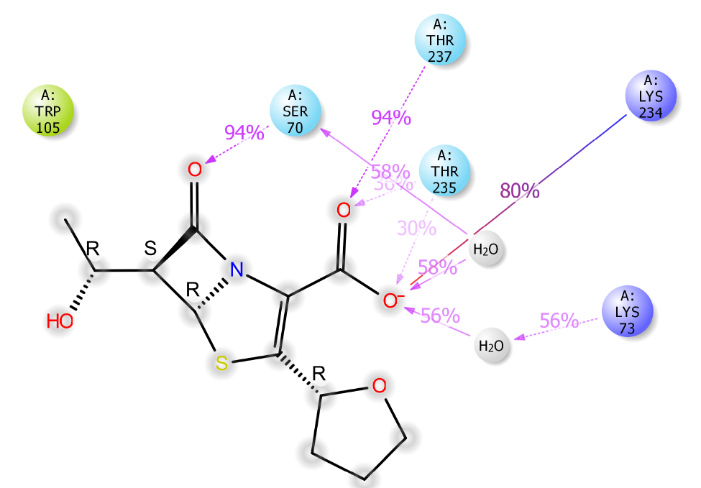  Co-ligand | |

Figure S2: A schematic of detailed ligand atom interactions with the protein residues. Interactions that occur more than 30.0% of the simulation time in the selected trajectory (0.00 through 150.00 ns), are shown.
